# SARFIMA model prediction for infectious diseases: application to hemorrhagic fever with renal syndrome and comparing with SARIMA

**DOI:** 10.1186/s12874-020-01130-8

**Published:** 2020-09-29

**Authors:** Chang Qi, Dandan Zhang, Yuchen Zhu, Lili Liu, Chunyu Li, Zhiqiang Wang, Xiujun Li

**Affiliations:** 1 Department of Biostatistics, School of Public Health, Cheeloo College of Medicine, Shandong University, Jinan, China; 2 Institute of Infectious Disease Control and Prevention, Shandong Center for Disease Control and Prevention, Jinan, China

**Keywords:** Seasonal autoregressive fractionally integrated moving average model, Seasonal autoregressive integrated moving average model, Hemorrhagic fever with renal syndrome, Goodness of fit, Prediction

## Abstract

**Background:**

The early warning model of infectious diseases plays a key role in prevention and control. This study aims to using seasonal autoregressive fractionally integrated moving average (SARFIMA) model to predict the incidence of hemorrhagic fever with renal syndrome (HFRS) and comparing with seasonal autoregressive integrated moving average (SARIMA) model to evaluate its prediction effect.

**Methods:**

Data on notified HFRS cases in Weifang city, Shandong Province were collected from the official website and Shandong Center for Disease Control and Prevention between January 1, 2005 and December 31, 2018. The SARFIMA model considering both the short memory and long memory was performed to fit and predict the HFRS series. Besides, we compared accuracy of fit and prediction between SARFIMA and SARIMA which was used widely in infectious diseases.

**Results:**

Model assessments indicated that the SARFIMA model has better goodness of fit (SARFIMA (1, 0.11, 2)(1, 0, 1)_12_: Akaike information criterion (AIC):-631.31; SARIMA (1, 0, 2)(1, 1, 1)_12_: AIC: − 227.32) and better predictive ability than the SARIMA model (SARFIMA: root mean square error (RMSE):0.058; SARIMA: RMSE: 0.090).

**Conclusions:**

The SARFIMA model produces superior forecast performance than the SARIMA model for HFRS. Hence, the SARFIMA model may help to improve the forecast of monthly HFRS incidence based on a long-range dataset.

## Background

The incidence of infectious diseases is subject to many factors, and there are intricate connections between the influencing factors. In recent years, many studies have explored the relationship between meteorological factors and infectious diseases [1–4]. However, the impact of meteorological factors account for only a small proportion on infectious diseases [1], because there are many potential unknown factors. It is especially important to establish a dynamic model of time series according to its own variation to predict and warn infectious diseases.

Time series analysis and modeling is widely used for studying temporal changes in the incidence of infectious diseases to forecast future trends [2, 5, 6]. Seasonal autoregressive integrated moving average (SARIMA) model has been used to fit and predict epidemics of many infectious diseases, such as cryptosporidiosis [7], scrub typhus [8], and bacterial foodborne diseases [9], and so on [10, 11]. The data preparation and model operation for SARIMA model are relatively simple and easy to perform [12], and the prediction results are accurate. Thereby, it is usually used to predict short-term fluctuations of infectious diseases. Compared to the SARIMA which is an integer order model, the seasonal autoregressive fractionally integrated moving average (SARFIMA) model considering both the short memory and long memory may be more accurate when modeling the infectious diseases data possessing the long memory property [13, 14]. Furthermore, the SARFIMA is as simple and easy as the SARIMA to perform in R software now.

In many time series, although the correlation between long-range observations are small, they should not be ignored [13]. The ARFIMA is given by Granger and Joyeux (1980) [15], and the extension, SARFIMA, was put forward by Porter-Hudak (1990) [16]. Any pure ARMA stationary time series can be considered a short memory series. Augmenting the standard ARMA model with a long memory component leads to the ARFIMA model. A series possessing long memory has an autocorrelation function (ACF) decaying more slowly than the geometric decay possessed by short memory processes, what is called hyperbolic decay (HD). Using first-order difference instead of fractional-order difference for a series exhibits long memory will lead to over-difference [15], and many useful features in the original series will be discarded, which will cause deviation in parameter estimation and modeling. The surveys of long memory models, which developed in hydrology, meteorology and geophysics [17] have not been widely applied in infectious diseases.

Our study applied the SARFIMA model to monthly HFRS incidence series mixing short memory (short-range dependence) and long memory (long-range dependence) for more accurate estimation. HFRS is a natural epidemic disease and remains a serious public health problem. There may be as many as 150,000 cases each year [18]. Moreover, the number of countries reporting human cases of HFRS is still on the rise [19]. Weifang city, which is located in northeastern China, is one of the most seriously affected areas since the first case of HFRS was reported in 1974. The better prediction of HFRS emergence can potentially reduce the effects of infections on humans. Therefore, comparing the prediction ability of SARFIMA and SARIMA models, and applying the better model to predict the trends for HFRS, conduce to provide important support for studying in the disease.

## Methods

### Model: SARIMA model and SARFIMA model

SARIMA models are useful for modeling seasonal time series [20], and it expressed as

1 $$ {\varnothing}_p(B){\Phi}_P\left({B}^s\right){\left(1-B\right)}^d{\left(1-{B}^s\right)}^D{x}_t={\theta}_q(B){\Theta}_Q\left({B}^s\right){\varepsilon}_t $$

Where *B* is the backward operator, *x*_*t*_ expresses series, *ε*_*t*_ is a white noise process, and *s* is the seasonal period, e.g., *s* = 12 for monthly series. The values of *d* are restricted to zero when the series modeled is stationary and to be a positive integer when the series must be differenced to eliminate nonstationary [17]. ∅_*p*_(*B*) is the nonseasonal AR operator of order *p*, and *θ*_*q*_(*B*) is the nonseasonal MA operator of order *q*. Φ_*P*_(*B*^*s*^) and Θ_*Q*_(*B*^*s*^) is the seasonal AR and MA operator, respectively. This model is often called a multiplicative SARIMA model, because the operators in the function are multiplied together rather than summed.

SARFIMA model allows for series to be fractionally integrated, generalizing the integer order of integration of the SARIMA model to allow the *d* parameter to take on fractional values [21]. If a series exhibits long memory, it is neither stationary (*I* (0)) nor is it a unit root (*I* (1)) process; the series is an *I*(*d*) process. Consider the following model:

2 $$ {\left(1-{B}^s\right)}^d{x}_t={\varepsilon}_t $$

where *d* is the fractionally differenced component and lies in (−0.5, 0.5). The model (2) is a direct seasonal analogue of the simple fractional differenced model:

3 $$ {\left(1-B\right)}^d{x}_t={\varepsilon}_t $$

The generalization of (2) to an ARMA model with a fractionally differenced seasonal component, namely, a SARFIMA model can be expressed as:

4 $$ {\left(1-{B}^s\right)}^d\Omega\ (B){x}_t=\Theta (B){\varepsilon}_t. $$

Where Ω (*B*) and Θ(*B*) are autoregressive and moving average polynomials, respectively (each including seasonal components). The restriction of *d* to take only integer values would simplify to an SARIMA model. For a stationary process, *d* varies between − 0.5 and 0.5, with *d* = 0 indicating short memory, − 0.5 < *d* < 0 indicating intermediate memory, and 0 < *d* < 0.5 indicating long memory [22].

For ARFIMA (*p*, *d* *, *q*), where *d* * = *d* + *d*_*f*_. Most commonly, *d*_*f*_∈ (− 0.5, 0.5) is the fractional part, and *d*≥ 0 always is the integer part. The Hurst exponent (*H*) is a measure of long memory of time series [23]. It relates to the autocorrelations of the time series and the rate at which these values decrease as the lag increases. The relationship between *d*_*f*_ and *H* is: *d*_*f*_ = *H* − 0.5; if *H* > 0.5, it would indicate a long-memory time series; if *H* < 0.5, it can be considered as an intermediate-memory time series. When *H* = 0.5, it would indicate a random walk. The statistical efficient model estimation is based on the method of maximum likelihood. For general long-memory time series models, this method has been shown to be asymptotically efficient [24].

### Data

The monthly HFRS reported data between 2005 to 2018 in Weifang city was obtained from Health Commission of Shandong Province (http://wsjkw.shandong.gov.cn/) and Health Commission of Weifang (http://wsjkw.weifang.gov.cn/) and Shandong Center for Disease Control and Prevention. The diagnostic criteria of HFRS was the Diagnostic Standards for Epidemic Hemorrhagic Fever (WS278–2008) (http://www.nhc.gov.cn/wjw/s9491/200802/39043.shtml). The criteria remained consistent during the study period. The HFRS incidence were calculated by the disease reported data and population size in Weifang city. The annual population size from 2005 to 2018 was extracted from Shandong Statistical Yearbook [25].

### Data analysis

For constructing and validating models, the data was divided into two datasets. The data from January 2005 to December 2017 was used to build models, and the data between January to December 2018 was regarded as the prediction set.

#### Construction of the SARIMA model

The SARIMA model requires a stationary time series. First, we drew the time series plot of the monthly HFRS incidence. We checked stationarity and seasonality by augmented Dickey-Fuller (ADF) test and seasonal decomposition. The model used to decomposition is: *Y*_*t*_ = *T*_*t*_ + *S*_*t*_ + *e*_*t*_. The function first determined the trend component using a moving average and removed it from the time series. Then, the seasonal component was computed by averaging for each time unit over all periods. Finally, the remainder component was determined by removing trend and seasonal component from the original time series. If the series is not stationary, it should be converted into a stationary series by difference (first-order difference or seasonal difference). We depicted the autocorrelation function (ACF) and partial autocorrelation function (PACF) plots to determine the order of model. The ACF plot shows the correlation of the series with itself at different lags, and the PACF plot shows the amount of autocorrelation at lag *k* that is not explained by lower-order autocorrelations. We selected the optimal SARIMA model with the lowest value in Akaike information criterion (AIC) from the candidate established models and used model diagnostic plots with Ljung-Box portmanteau test to assess the models.

#### Construction of the SARFIMA model

The corrected R/S Hurst exponent was computed to test the long memory of the monthly HFRS incidence series [26]. If the series has strong enough long memory, the SARFIMA model can be constructed. The order (*p*, *d*, *q*) and the seasonal components (*P*, *D*, *Q*) of the model was specified same as the SARIMA above. The SARFIMA fitting function based on the assumption that there will be multiple modes. That is, the fitting function will start the optimizations at multiple starting points. There can be more than one mode for time series models, and the best mode of the SARFIMA fits was found by means of log-likelihood value [27].

After fitting models, we examine the chosen model for possible inadequacies which could invalidate the model. The residual plot and Ljung-Box test were determined to evaluate the goodness of fit. Finally, we applied the best model to forecasting the monthly incidence of HFRS in the last year of dataset.

#### Comparison between the two models for performance

To evaluate forecast accuracy as well as to compare among two models, we have used the root mean square error (RMSE), the mean absolute error (MAE) and the mean absolute percentage error (MAPE) [28, 29].

All analyses were conducted with R (version 3.6.0), modeling with “arfima” and “ts” packages for SARFIMA and SARIMA models respectively.

## Results

### Description of time series

**Fig. 1:**
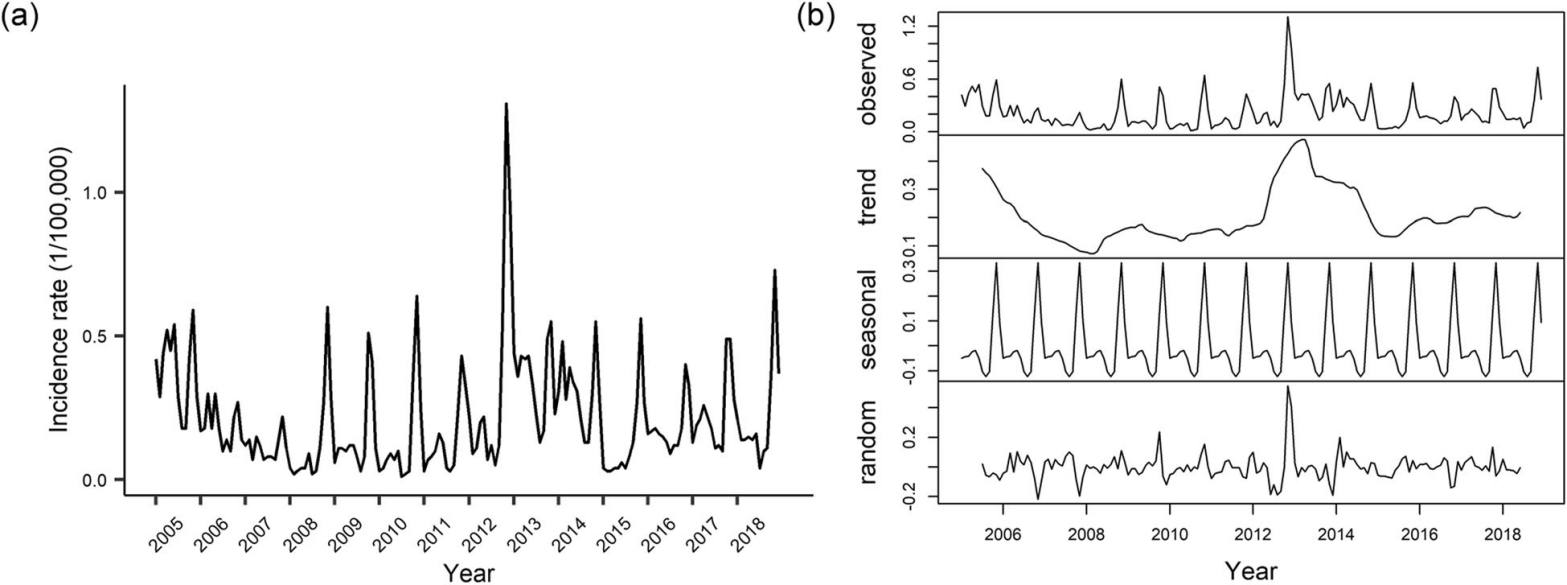
The monthly HFRS incidence time series **a** and seasonal decomposition **b** in Weifang city, Shandong Province, 2005–2018

During from 2005 to 2018, a total of 3302 HFRS cases were reported in Weifang city. There was a median of 14 (interquartile range: 8–26) cases every month. Figure 1 shows the monthly incidence trend during the study period, with a monthly incidence from 0.01 (1/100,000, minimum in July 2010) to 1.31 (1/100,000, maximum in November 2012). The series shows a noticeable seasonal pattern since HFRS possess two incidence peaks each year (April to June was the small peak and October to January was the predominant peak). We decomposed the time series, and the seasonality is clearly visible for HFRS time series.

### SARIMA model

**Fig. 2:**
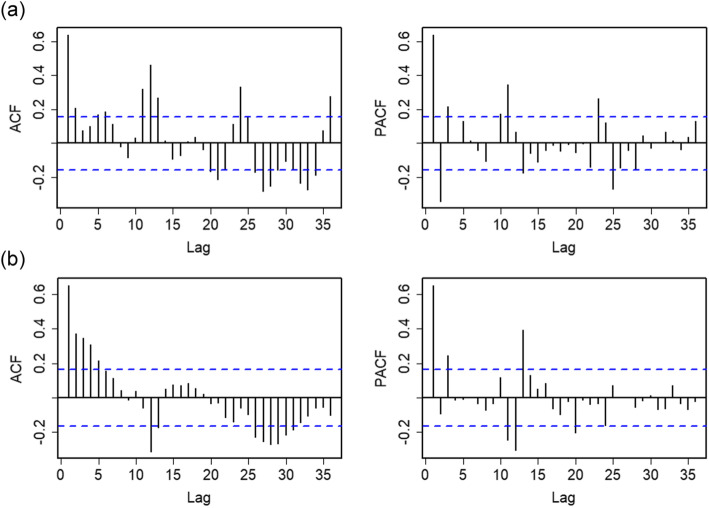
ACF and PACF plots of the original series **a** and seasonal differenced series **b** for HFRS time series in Weifang city, Shandong Province, 2005–2017

The ADF test indicates that the original series was stationary (Dickey-Fuller = − 3.95, *P* = 0.01), do not need for trend difference. However, the seasonal decomposition plot shows that the HFRS monthly incidence has evident seasonal pattern (Fig. 1b). The ACF and PACF plots of original series clearly display slow decay at the seasonal lags (Fig. 2a). Therefore, a lag-12 (subtract the observations after a lag of 12 periods) difference is used to remove the features of seasonality (Fig. S1). The ACF and PACF of seasonal differenced series have some significant spikes (Fig. 2b). Thus, the order of AR(*p*) and MA(*q*) was identified. Of all the tested models showed in Table S1 and Fig. S2, a SARIMA (1, 0, 2)(1, 1, 1)_12_ model was found to best fit the data (AIC =  − 227.32). This SARIMA model is (1 − 0.910*B*)(1 + 0.085*B*^12^)(1 + 0.999*B*^12^)*x*_*t*_ = (1 + 0.103*B* + 0.286*B*^2^)*ε*_*t*_.

### SARFIMA model

The corrected R/S Hurst exponent (*H* = 0.81, more than 0.5) indicated that the HFRS series exists strong long memory. The ACF of seasonal differenced HFRS series exhibits a slow decay pattern that is typical of a fractional model. The SARFIMA model was constructed based on the appropriate order of AR(*p*) and MA(*q*). The nonseasonal and seasonal fractional difference parameter were computed, and the best mode of a SARFIMA fit was found by removing modes with lower log-likelihoods (SARFIMA (1, 0.11, 2)(1, 0, 1)_12_, AIC = − 631.31). The SARFIMA model is (1 − 0.919*B*−)(1 + 0.973*B*^12^)(1 + 0.939*B*)^0.114^*x*_*t*_ = (1 − 0.459*B* − 0.327*B*^2^)*ε*_*t*_.

**Fig. 3:**
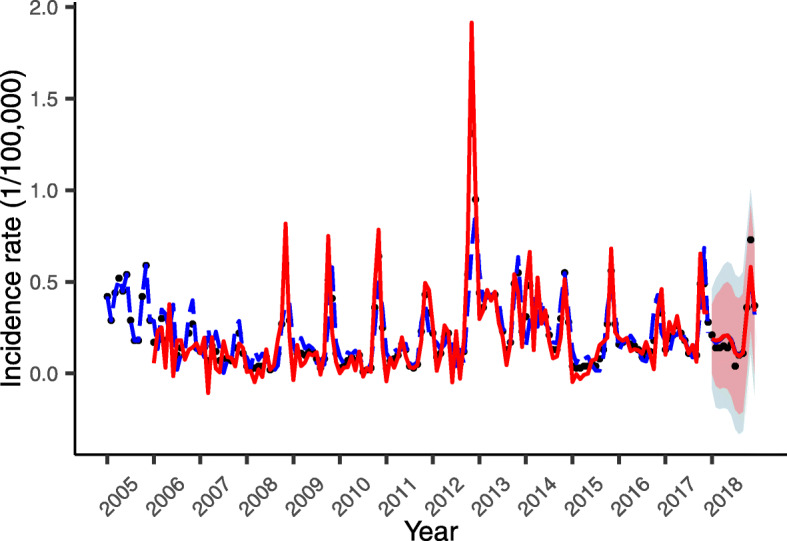
Fitting and forecast results of models. Black points indicate the real observations and lines indicate the simulated time series (SARFIMA: red solid line; SARIMA: blue dotted line). The shaded regions indicate 95% confidence intervals

The residual plots and the Ljung-Box tests of SARIMA and SARFIMA showed that the residuals are white noise (Fig. S3 and Table S2). The forecast results of models were showed in Fig. 3. As can be seen from the figure, the prediction trend of SARFIMA model was closer to the real values than SARIMA. The 95% confidence interval of SARFIMA model was narrower than SARIMA, and its interval included all the actual values. Therefore, the fractional differenced model did quite well compare to the integer differenced model. Table 1 gives the forecasting accuracy of two models for the HFRS series. The SARFIMA model has lower values for RMSE, MAE and MAPE, which means the SARFIMA is more accurate.

**Table 1:** Accuracy measures for SARIMA and SARFIMA models

	RMSE	MAE	MAPE
SARIMA(1, 0, 2)(1, 1, 1)_12_	0.090	0.059	46.704
SARFIMA(1, 0.11, 2)(1, 0, 1)_12_	0.058	0.044	32.549

## Discussion

Time series analysis is a method of applying mathematical models to represent the correlation of data and predicting future development trends. The SARIMA model is a common time series analysis method and is widely used to detect outbreaks of infectious diseases and predict their epidemics. In this study, we discussed the effect of SARFIMA model applied to HFRS series and compared with the SARIMA model. The notable fluctuations of monthly HFRS incidence were observed in the study period, and long memory of it was measured. We analyzed these features and constructed predictive models.

It is generally believed that based on large enough observations, that is, more than 50 data, the time series model constructed can obtain satisfactory prediction results. For SARFIMA model, the data selection should consider two points: First, the sample size of data is large enough [16]. For example, the simulation results were reported by Robinson [30] with a sample size of 64, and the series used by Chambers [31] were 152 quarterly observations. Whereas Braun [32] suggesting that time series with long memory should consist of around 500 observations. Second, the long-term memory of time series should be strong. For instance, the long memory of 5-year HFRS series extracted from our dataset is not strong enough (*H* = 0.48 < 0.5), and the sample size (*n* = 60) is not large enough. In our study, the length of monthly HFRS incidence data used to analysis was 168, and the time span of the series is form January 1, 2005 to December 31, 2018. The corrected R/S Hurst exponent displays the long memory of the HFRS series is strong. The results of model construction indicate that the chosen models fit the observations well, and the residual series were satisfied with white noises.

For the original data, the seasonal peak of the monthly HFRS incidence is obvious, indicating that the models should consider the seasonal components. For example, the prevailing HFRS occurred in October to January, and the incidence peaked in November. The plot of forecast results showed that the model prediction is consistent with it. The AIC values represent that the SARFIMA model considering the fractional difference outperform the SARIMA model in model fitting. All of three forecast accuracy measures of SARFIMA model are smaller than SARIMA model, so the predictive effects of SARFIMA are obviously better than SARIMA. In addition, the 95% confidence interval of SARFIMA is narrower than SARIMA. Generally speaking, SARFIMA model has a better effect on predicting the trend of monthly HFRS incidence series which possesses long-memory and short-memory process. Therefore, on the basis of a combination of best statistical and accuracy effect, the SARFIMA model should be chosen in preference to the SARIMA model, although SARIMA is relatively parsimony [33].

Granger and Joyeux [15] have reported that ARFIMA may give better longer-term forecasts. Therefore, we conducted a long-range prediction. The results of fit and forecast were showed in the Fig. S4. Nevertheless, the long-term predictions, take 3-year forecast as example, with the increasing steps of prediction, errors on the prediction are increasing. The prediction accuracy of SARFIMA (RMSE: 0.084) is comparable to SARIMA (RMSE: 0.098). The predicted values of more than 12 steps (1 year) is lower (deviation) from the true values. The possible reasons are as follows: First, the accuracy of a model estimated from historical data depends on the quality of the input values. The longer the time to predict, the less accurate the prediction becomes. Second, there are more changes components on long-term scales, because infectious diseases are affected by many factors [34].

This work shows the usefulness of SARFIMA in modeling the HFRS series. With the development of infectious disease surveillance system, the long-term datasets were more easily to access. In this case, there is a need for a new model that is capable of analyzing the long-term memory of datasets to improve the precision of the predictions. The application of SARFIMA to a wider range of infectious disease data is worth further investigation.

We also have performed the SARFIMA to other seasonal infectious disease to see how useful the model will be (Fig. S5, S6, S7 and Table S3). The number of observations in the mumps series is 72, and the long memory is strong (*H* = 0.82), which is suitable for analysis with SARFIMA. Therefore, SAFRIMA performs superior prediction than SARIMA.

There are several limitations in our study. First, the occurrence and prevalence of infectious diseases are affected by multiple factors such as natural factors, climate and human environment improvement, urban construction and other social factors. The time series model often consider the characteristics of the series itself but do not incorporate these factors into the model. Second, we only took several infectious diseases into account in this study, and the generalizability for the superior prediction of SARFIMA model still needs further research to prove. Although we have not illustrated it here, ARFIMA may also fit ARFIMA-X models with additional exogenous regressors, which can be further explored in future research.

## Conclusions

We explore the value of the SARFIMA model in the epidemic prediction research by means of comparison between SARFIMA and SARIMA models. Understanding and incorporating the long memory features will provide more accurate modeling and prediction for infectious diseases. In this respect, the SARFIMA model for forecasting the monthly incidence of HFRS are better than the SARIMA model.

## Supplementary information

**Additional file 1.**

## Data Availability

In this paper, we used the secondary data from Health Commission of Shandong Province (http://wsjkw.shandong.gov.cn/) and Health Commission of Weifang (http://wsjkw.weifang.gov.cn/). Besides, our co-author in Shandong Center for Disease Control and Prevention provided some data.

## Abbreviations

SARFIMA: Seasonal autoregressive fractionally integrated moving average
SARIMA: Seasonal autoregressive integrated moving average
HFRS: Hemorrhagic fever with renal syndrome
AIC: Akaike information criterion
RMSE: Root mean square error
ACF: Autocorrelation function
PACF: Partial autocorrelation function
HD: Hyperbolic decay
MAE: Mean absolute error
MAPE: Mean absolute percentage error
ADF: Augmented Dickey-Fuller
