# Supplementary material for: SARFIMA model prediction for infectious diseases: application to hemorrhagic fever with renal syndrome and comparing with SARIMA
Source: BMC Med Res Methodol. 2020 Sep 29;20:243. doi: 10.1186/s12874-020-01130-8 (PMC7526348; doi:10.1186/s12874-020-01130-8)
Supplement: Supplementary file 1 — Additional file 1. [file 12874_2020_1130_MOESM1_ESM.pdf]

SARFIMA model prediction for infectious diseases: application to hemorrhagic fever with renal syndrome and comparing with SARIMA

Chang Qi[1], Dandan Zhang[1], Yuchen Zhu[1], Lili Liu[1], Chunyu Li[1], Zhiqiang Wang[2], Xiujun Li[1*]

Supplementary Materials

[1] Department of Biostatistics, School of Public Health, Cheeloo College of Medicine, Shandong University, Jinan, China
[2] Institute of Infectious Disease Control and Prevention, Shandong Center for Disease Control and Prevention, Jinan, China
[*] Correspondence: xjli@sdu.edu.cn

Part 1

SARFIMA model applied in hemorrhagic fever with renal syndrome (HFRS)

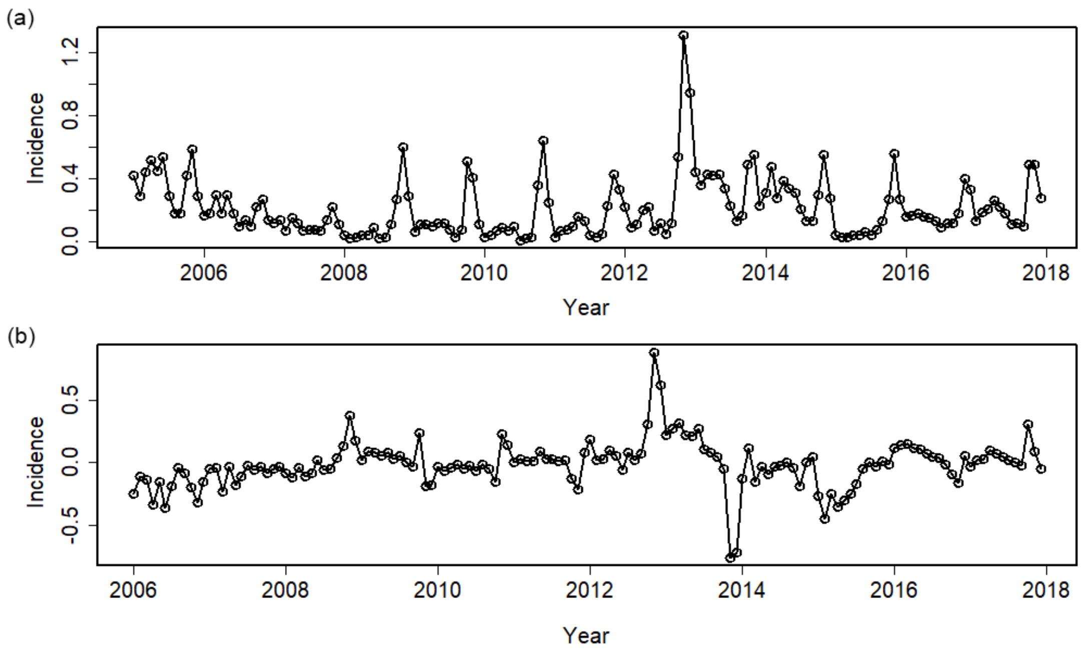

Fig S1. Original series (a) and seasonal differenced series (b) for HFRS time series in Weifang city, Shandong Province, 2005-2017

Table S1. Detailed information for the candidate SARIMA models

SARIMA Model	σ^2	Log likelihood	AIC	BIC
$(2, 0, 2)(1, 1, 0)_{12}$	0.013	104.02	-196.04	-178.22
$(2, 0, 2)(1, 1, 1)_{12}$	0.009	119.93	-225.85	-205.07
$(2, 0, 2)(2, 1, 1)_{12}$	0.009	120.00	-223.99	-200.24
$(2, 0, 2)(1, 1, 2)_{12}$	0.009	120.69	-225.38	-201.62
$(1, 0, 2)(1, 1, 1)_{12}$	0.009	119.66	**-227.32**	-209.50
$(1, 0, 1)(1, 1, 1)_{12}$	0.009	116.69	-223.38	-208.53

(a)

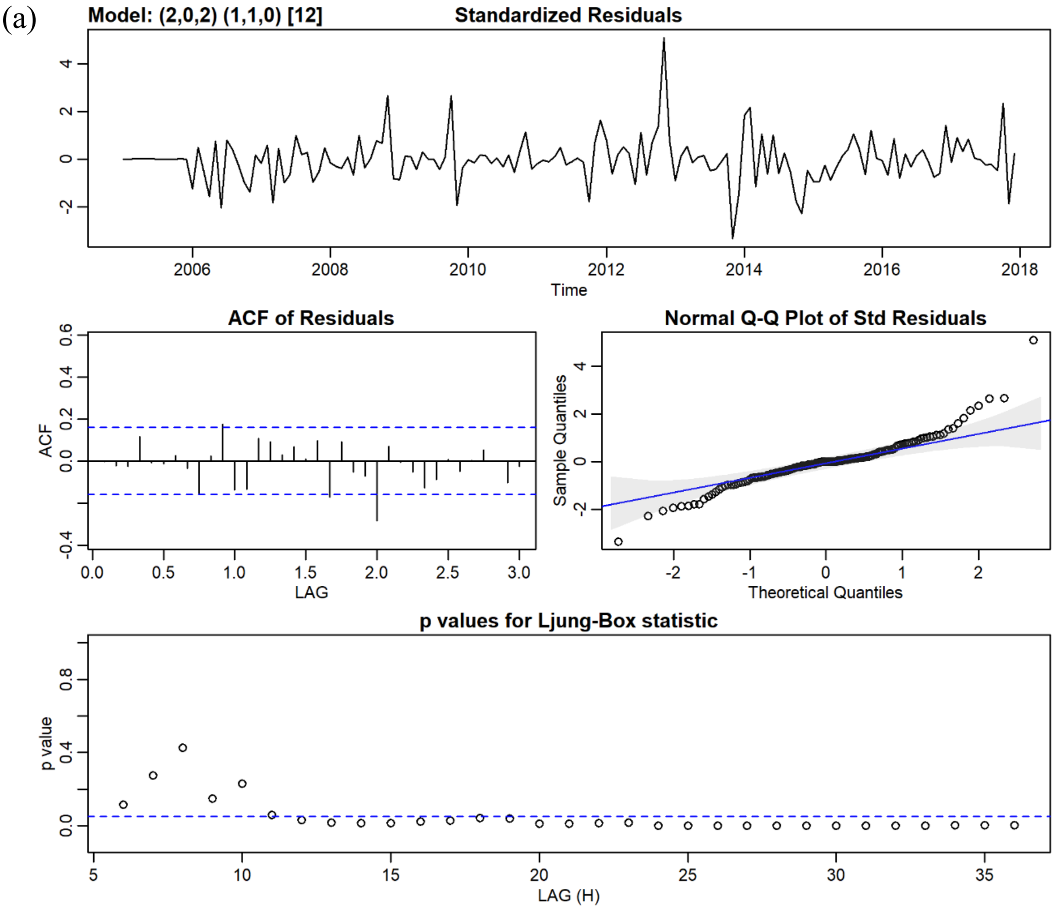
Model: (2,0,2) (1,1,0) [12]
Standardized Residuals
Time
ACF of Residuals
ACF
LAG
Normal Q-Q Plot of Std Residuals
Sample Quantiles
Theoretical Quantiles
p values for Ljung-Box statistic
p value
LAG (H)

(b)

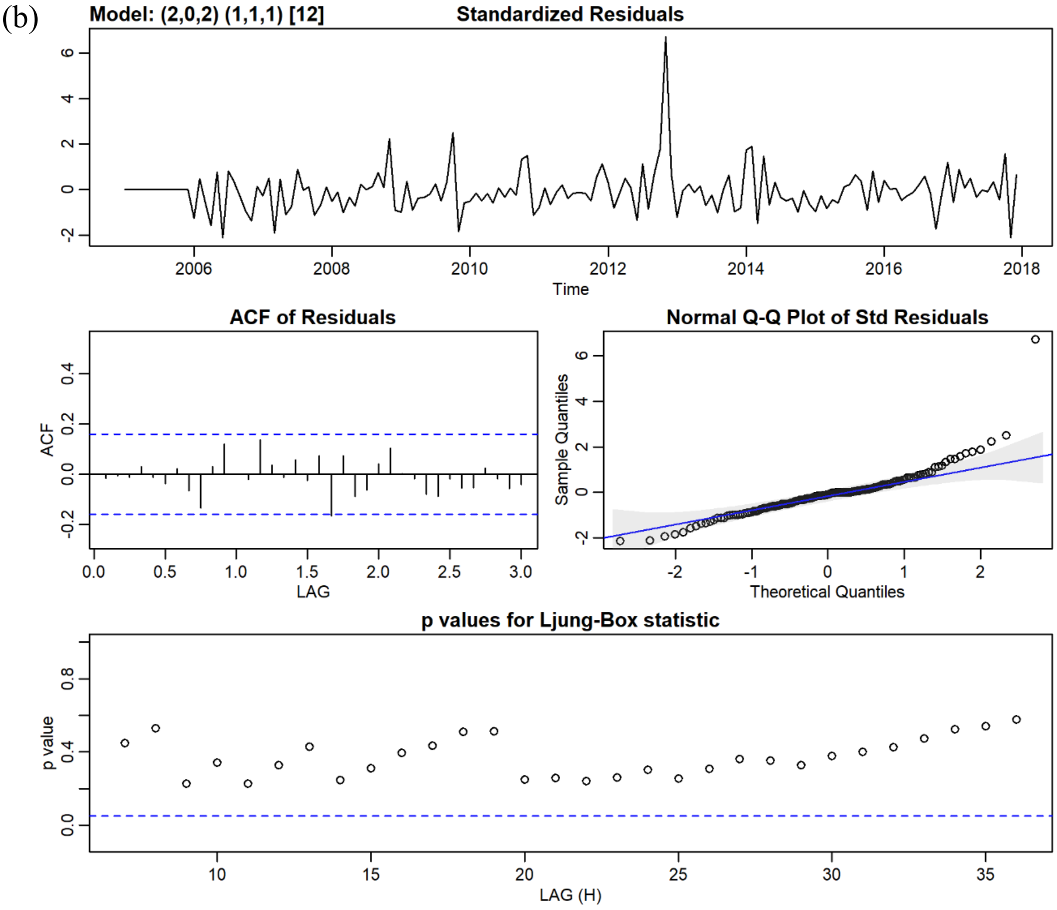
Model: (2,0,2) (1,1,1) [12]
Standardized Residuals
Time
ACF of Residuals
ACF
LAG
Normal Q-Q Plot of Std Residuals
Sample Quantiles
Theoretical Quantiles
p values for Ljung-Box statistic
p value
LAG (H)

(c)

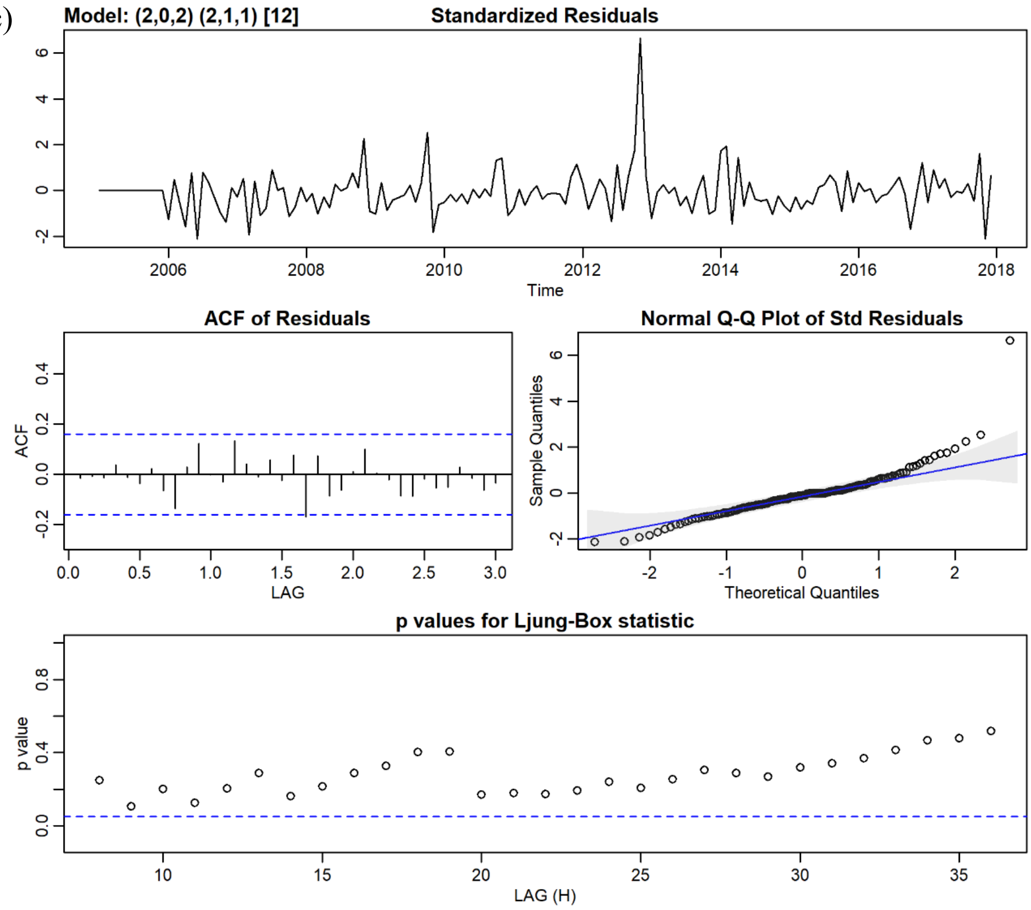
Model: (2,0,2) (2,1,1) [12]
Standardized Residuals
Time
ACF of Residuals
ACF
LAG
Normal Q-Q Plot of Std Residuals
Sample Quantiles
Theoretical Quantiles
p values for Ljung-Box statistic
p value
LAG (H)

(d)

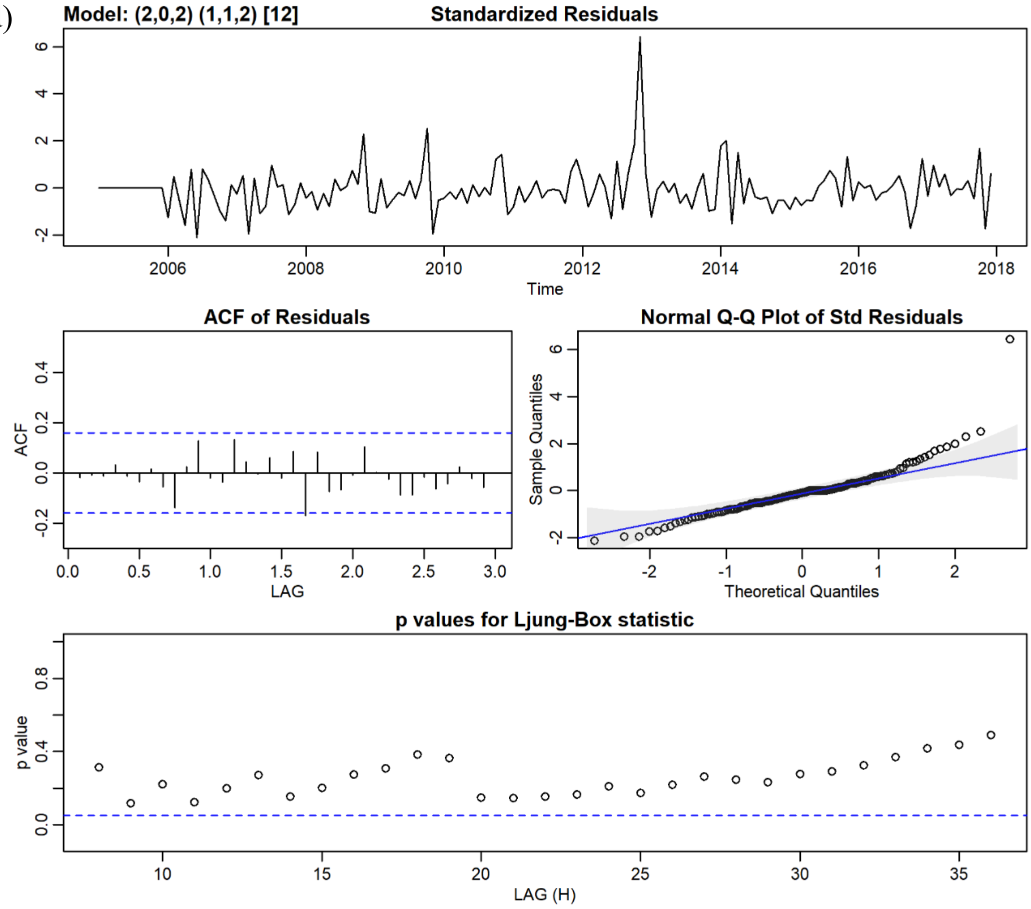
Model: (2,0,2) (1,1,2) [12]
Standardized Residuals
Time
ACF of Residuals
ACF
LAG
Normal Q-Q Plot of Std Residuals
Sample Quantiles
Theoretical Quantiles
p values for Ljung-Box statistic
p value
LAG (H)

(e)

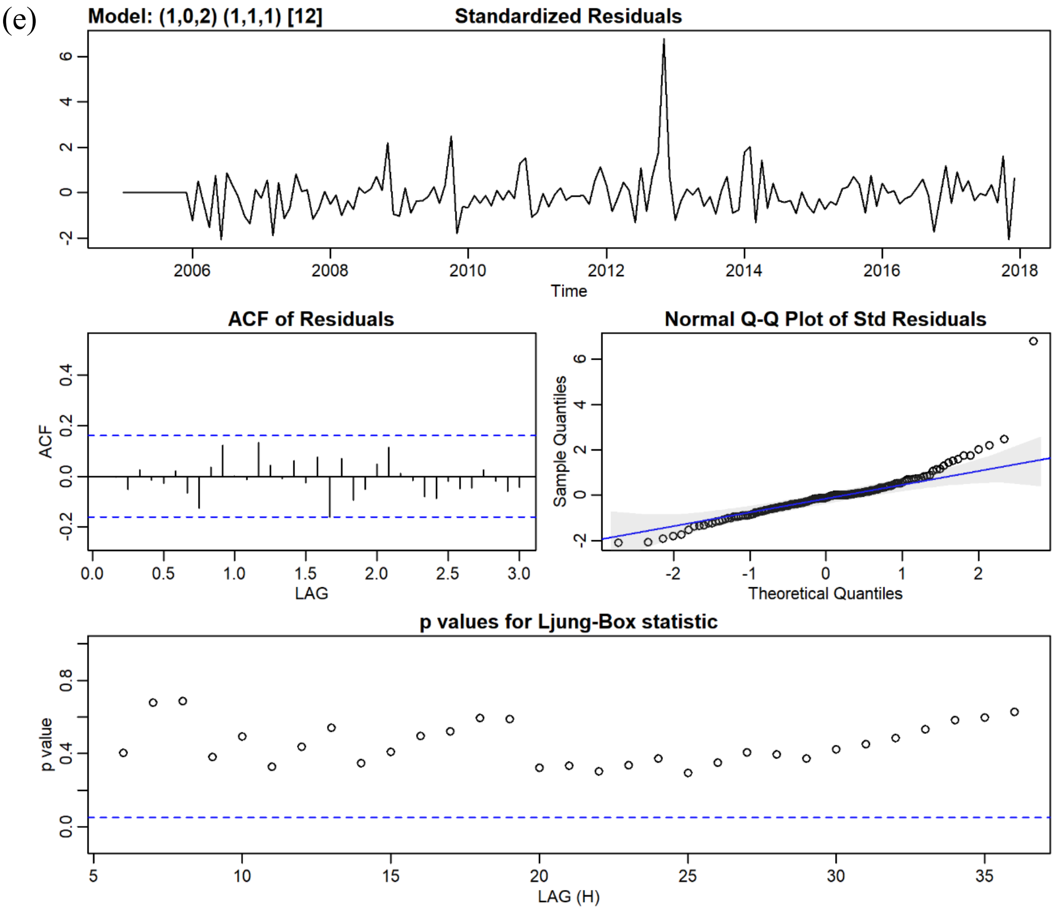

(f)

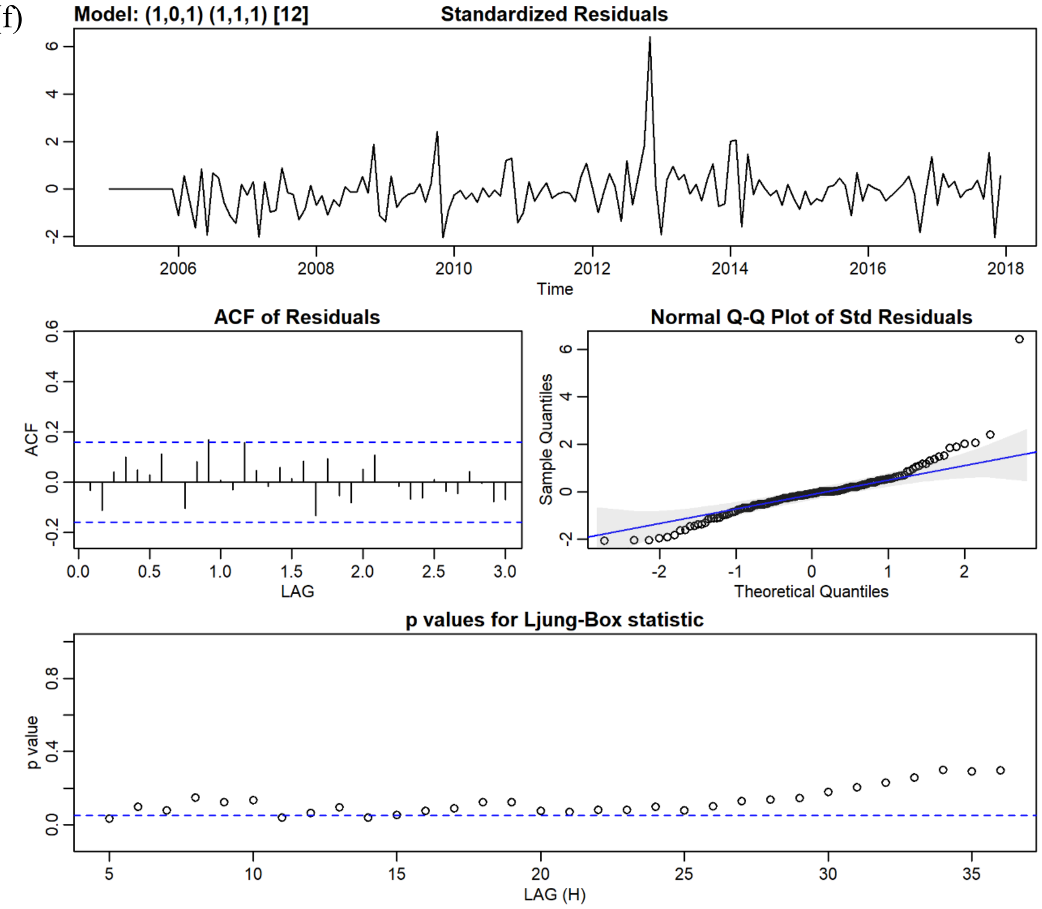

Fig S2. Model diagnostics for the SARIMA models fitted to the monthly HFRS incidence series from January 1, 2005 to December 31, 2017

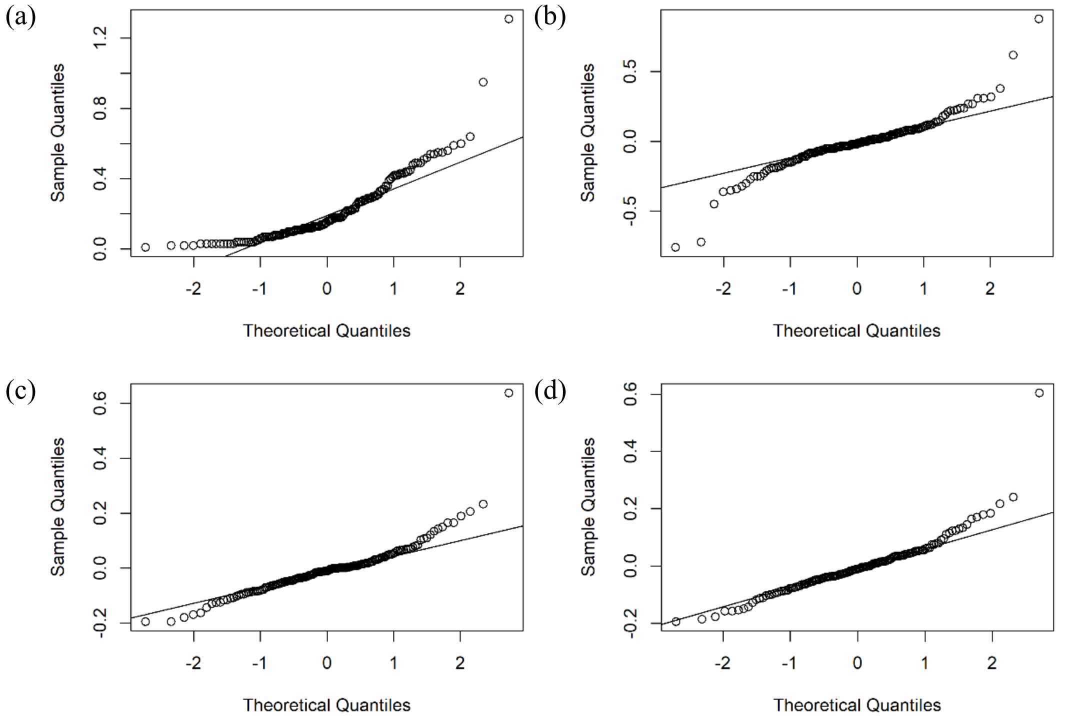

Fig. S3 The residual plots of original series (a), seasonal differenced series (b), SARIMA(1, 0, 2)(1, 1, 1)$_{12}$ model (c) and SARFIMA(1, 0.11, 2)(1, 0, 1)$_{12}$ model (d)

Table S2 The optimal SARIMA and SARFIMA models and the Ljung-Box test results

Model	χ^2	*P* value
SARIMA(1, 0, 2)(1, 1, 1)$_{12}$	3.216×10^{-4}	0.986
SARFIMA(1, 0.11, 2)(1, 0, 1)$_{12}$	0.115	0.735

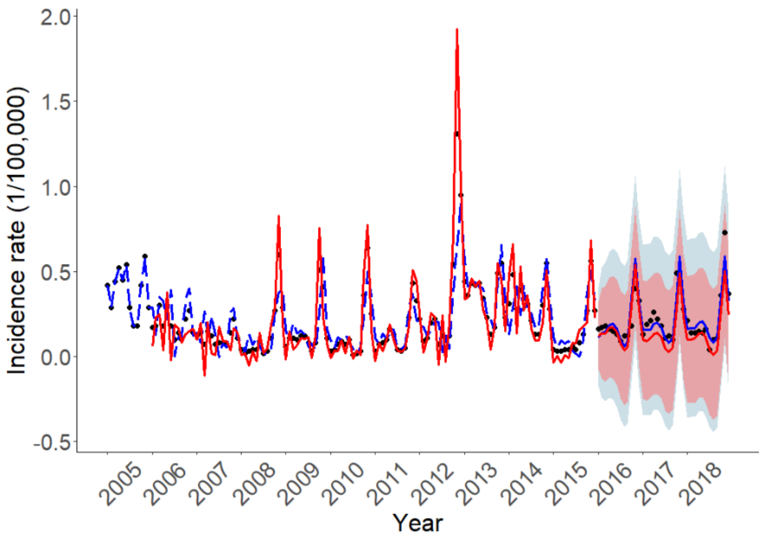

Fig. S4 3-year forecast for HFRS of SARIMA and SARFIMA. Black points indicate the real observations and lines indicate the simulated time series (SARFIMA: red solid line; SARIMA: blue dotted line). The shaded regions indicate 95% confidence intervals

Part 2

SARFIMA model applied in other infectious diseases

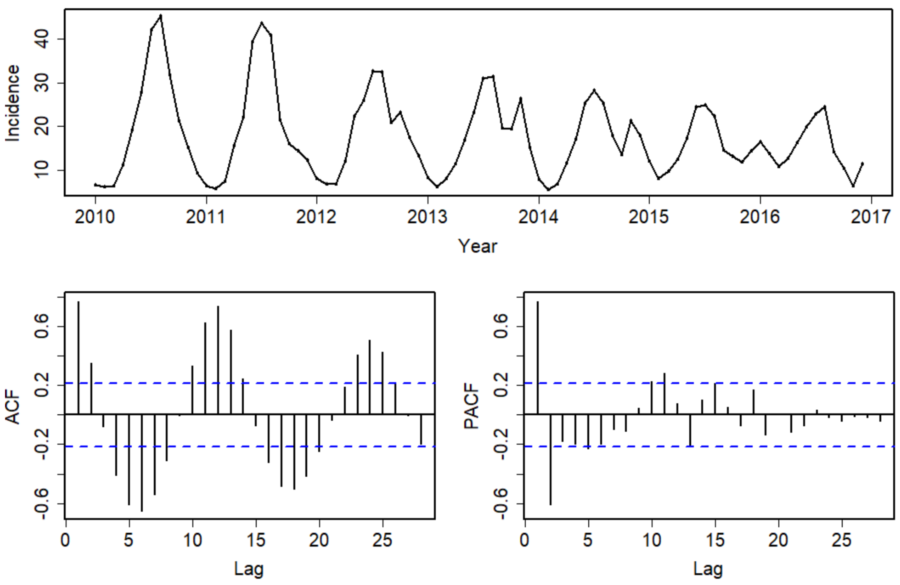

Fig. S5 Time series along with ACF and PACF plots for mumps from 2010 to 2016 in Beijing. The data was collected from the web-based reporting system for notifiable infectious diseases

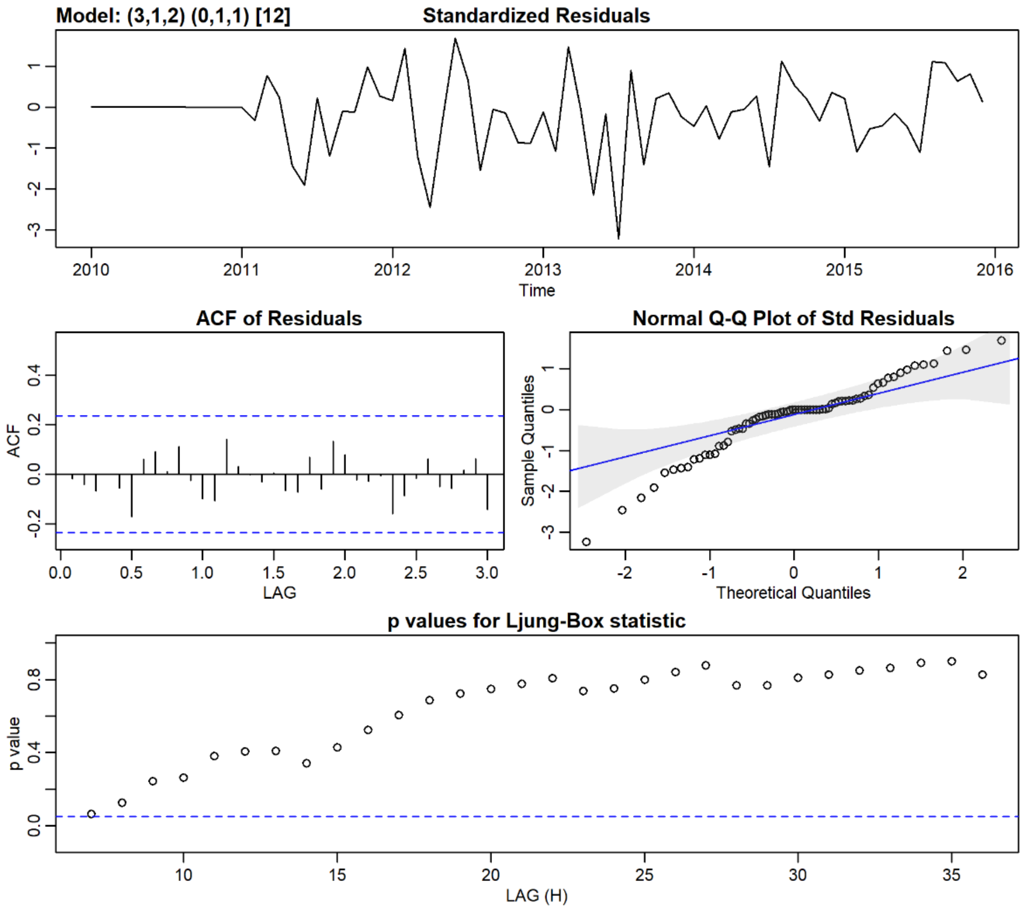

Fig S6. Model diagnostics for the SARIMA models fitted to the monthly mumps incidence series from January 1, 2010 to December 31, 2015

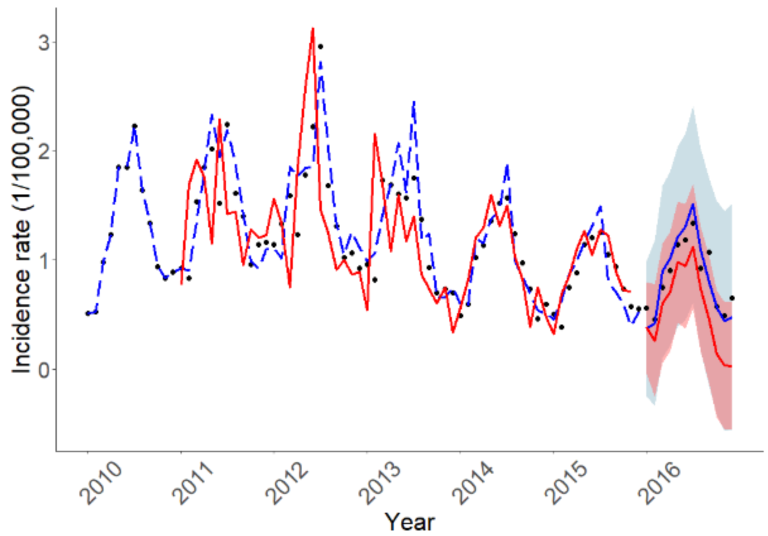

Fig. S7 Fitting and forecast results of SARIMA and SARFIMA models for Mumps. Black points indicate the real observations and lines indicate the simulated time series (SARFIMA: red solid line; SARIMA: blue dotted line). The shaded regions indicate 95% confidence intervals

Table S3 Accuracy measures of SARIMA and SARFIMA models for mumps

	RMSE	MAE
SARIMA(3, 1, 2)(0, 1, 1)$_{12}$	2.829	2.012
SARFIMA(3, 0.32, 2)(0, 0.54, 1)$_{12}$	0.349	0.304
